# 5-Aminolevulinic acid increases boronophenylalanine uptake into glioma stem cells and may sensitize malignant glioma to boron neutron capture therapy

**DOI:** 10.1038/s41598-023-37296-6

**Published:** 2023-06-22

**Authors:** Masao Fukumura, Naosuke Nonoguchi, Shinji Kawabata, Ryo Hiramatsu, Gen Futamura, Koji Takeuchi, Takuya Kanemitsu, Takushi Takata, Hiroki Tanaka, Minoru Suzuki, Oltea Sampetrean, Naokado Ikeda, Toshihiko Kuroiwa, Hideyuki Saya, Ichiro Nakano, Masahiko Wanibuchi

**Affiliations:** 1 Institute for Integrated Radiation and Nuclear Science, Kyoto University, Kumatori, Osaka Japan; 2 Division of Gene Regulation, Institute for Advanced Medical Research, Keio University School of Medicine, Tokyo, Japan; 3 Department of Neurosurgery, Tesseikai Neurosurgical Hospital, Shijonawate, Osaka Japan; 4 Department of Neurosurgery, University of Alabama at Birmingham, Birmingham, AL USA; 5 Present Address: Department of Neurosurgery, Osaka Medical and Pharmaceutical University, Takatsuki, Osaka 569-8686 Japan

**Keywords:** Stem cells, CNS cancer

## Abstract

Boron neutron capture therapy (BNCT) is a high-LET particle radiotherapy clinically tested for treating malignant gliomas. Boronophenylalanine (BPA), a boron-containing phenylalanine derivative, is selectively transported into tumor cells by amino acid transporters, making it an ideal agent for BNCT. In this study, we investigated whether the amino acid 5-aminolevulinic acid (ALA) could sensitize glioma stem cells (GSCs) to BNCT by enhancing the uptake of BPA. Using human and mouse GSC lines, pre-incubation with ALA increased the intracellular accumulation of BPA dose-dependent. We also conducted in vivo experiments by intracerebrally implanting HGG13 cells in mice and administering ALA orally 24 h before BPA administration (ALA + BPA-BNCT). The ALA preloading group increased the tumor boron concentration and improved the tumor/blood boron concentration ratio, resulting in improved survival compared to the BPA-BNCT group. Furthermore, we found that the expression of amino acid transporters was upregulated following ALA treatment both in vitro and in vivo, particularly for ATB^0,+^. This suggests that ALA may sensitize GSCs to BNCT by upregulating the expression of amino acid transporters, thereby enhancing the uptake of BPA and improving the effectiveness of BNCT. These findings have important implications for strategies to improve the sensitivity of malignant gliomas to BPA-BNCT.

## Introduction

Malignant gliomas are the most prevalent and lethal among intraparenchymal brain tumors, and glioblastomas (GBM) have the worst prognosis^1^. Presently, The standard treatment for malignant gliomas includes combined therapy with surgical resection, chemotherapy, and radiotherapy. Nevertheless, the median survival time (MST) of GBM patients is < 15 months because of tumor recurrence. Studies during the last decade suggest that malignant gliomas harbor self-renewing tumorigenic cancer stem cells, termed glioma stem cells (GSCs). GSCs are believed to be responsible for the therapeutic resistance of malignant gliomas and tumor recurrence^2^. Thus, treatment strategies targeting GSCs might help treat GBM^3^.

BNCT (Boron Neutron Capture Therapy) is a high-linear energy transfer (LET) particle radiation therapy that allows for the selective targeting of tumor cells. In the actual treatment, a drug containing boron-10 (^10^B) is first administered to the patient, followed by irradiation with epithermal neutron beams focused on the tumor lesion. In the cells where the drug is taken up, a neutron capture reaction occurs specifically with ^10^B, generating alpha particles. Alpha particles release all their energy within a short range of less than the size of a cell (approximately 10 μm), leading to a high-density ionization reaction with cell organelles and various intracellular molecules. In particular, complex double-strand breaks, which are difficult to repair, occur in DNA, resulting in a powerful cytotoxic effect. The ^10^B drug for BNCT is designed to selectively accumulate in malignant tumor cells, allowing for the preservation of normal brain cells with low radiation exposure^4^. Thus, BNCT is a selective radiotherapy targeting tumor cells with high boron concentrations.

The leading ^10^B drug currently used in clinical practice is boronophenylalanine (BPA), an analog of phenylalanine; BPA has already been approved for insurance coverage in Japan as the anti-neoplastic agent for BNCT, specifically for refractory head and neck cancer. BPA selectively accumulates in malignant tumor tissues via the amino acid transport system^5^. We have conducted clinical trials on BPA-mediated BNCT against malignant brain tumors, including gliomas, since 2002^6^, and we are currently performing a multicenter phase II clinical trial for accelerator-based BNCT to treat patients with recurrent GBM in hospitals^7^. Unfortunately, even with BNCT, the recurrence of malignant gliomas cannot be prevented. Therefore, in BNCT using BPA, increasing BPA concentration in GSCs might help target these intractable tumor-propagating cells.

5-Aminolevulinic acid (ALA) is a natural amino acid synthesized from glycine and succinyl-CoA in mammalian cells. Subsequently, via multiple enzymatic reactions, ALA is converted to porphyrin. Exogenous ALA, a drug approved by the FDA for photodynamic diagnosis (PDD), accumulates selectively in neoplastic cells^8^. As a result, excessive accumulation of protoporphyrin IX (PpIX), a photosensitive substance, occurs only in tumors. The fluorescence of this exogenous ALA-derived PpIX has been utilized for the PDD of tumors, including malignant gliomas^9^.

In our preliminary study, we observed that ALA significantly increases the intracellular concentration of phenylalanine in human and mouse GSCs (see Supplementary Fig. S1). We hypothesized that ALA treatment could increase BPA uptake into GSCs and enhance the therapeutic efficacy of BNCT. To test the hypothesis, we treated human and mouse GSC lines with ALA and BPA and measured the intracellular boron concentration. The effect of ALA on the expression of neutral amino acid transporters and Reactive Oxygen Species (ROS) regulatory genes was evaluated by qPCR. Finally, we evaluated the therapeutic efficacy of ALA pretreatment in BNCT using the mouse glioma model.

## Results

To show that ALA pretreatment enhances the therapeutic efficacy of BPA-based BNCT, we first confirmed that ALA (300 or 900 μΜ) pretreatment significantly increased the BPA uptake in GSCs (HGG13, HGG30, and TS) (Fig. 2a–c). We subsequently confirmed that the expression of ATB^0,+^, a neutral amino acid transporter involved in BPA uptake, was increased in GSCs (HGG13 and HGG30) (Fig. 3a,b). In a xenograft brain tumor model prepared by transplanting the HGG13 cells into nude mouse brains, oral administration of ALA significantly elevated the expression of ATB^0,+^ in the tumors (Fig. 4). In the same brain tumor model, the biodistribution of BPA was examined in the ALA-pretreated group and PBS-pretreated (control) group. The mean ^10^B concentration in the tumor, Tumor/Brain ratio of ^10^B, and Tumor/Blood ratio of ^10^B were higher in the ALA-pretreated group compared to the control group (23.2 vs. 20.7; *p* = 0.23, 5.5 vs. 4.7; *p* = 0.41, and 5.8 vs. 4.3; *p* = 0.03) (Table 1). Finally, in the BNCT study, the BNCT with ALA preloading group significantly prolonged survival compared to the BNCT alone group (MST: 25 days vs. 28 days; *p* = 0.02) (Fig. 5).

**Table 1:** Summary of boron biodistribution in HGG13 glioma-bearing mice.

Group	n	Boron concentration ± SD (μg ^10^B/g)			Ratios^a^	
		Tumor	Normal brain	Blood	T/Br	T/Bl
BPA/i.p.^b^	4	20.7 ± 1.6	4.4 ± 1.1	4.8 ± 0.4	4.7	4.3
ALA/p.o. + BPA/i.p.^b,c^	4	23.2 ± 2.8	4.2 ± 0.4	4.0 ± 0.9	5.5	5.8
					*p* = 0.41	*p* = 0.03

SD standard deviation, p.o. per os, i.p. intraperitoneal.

^a^ T/Bl indicates the tumor to blood boron concentration ratio.

^b^BPA was administered i.p. at a dose of 12 mg ^10^B/kg b.w. (= 250 mg BPA/kg b.w.)

^c^5-Aminolevulinic acid was administered orally at 80 mg/kg b.w. 24 h before BPA administration.

### BPA uptake in cultured GSCs

**Figure 1:**
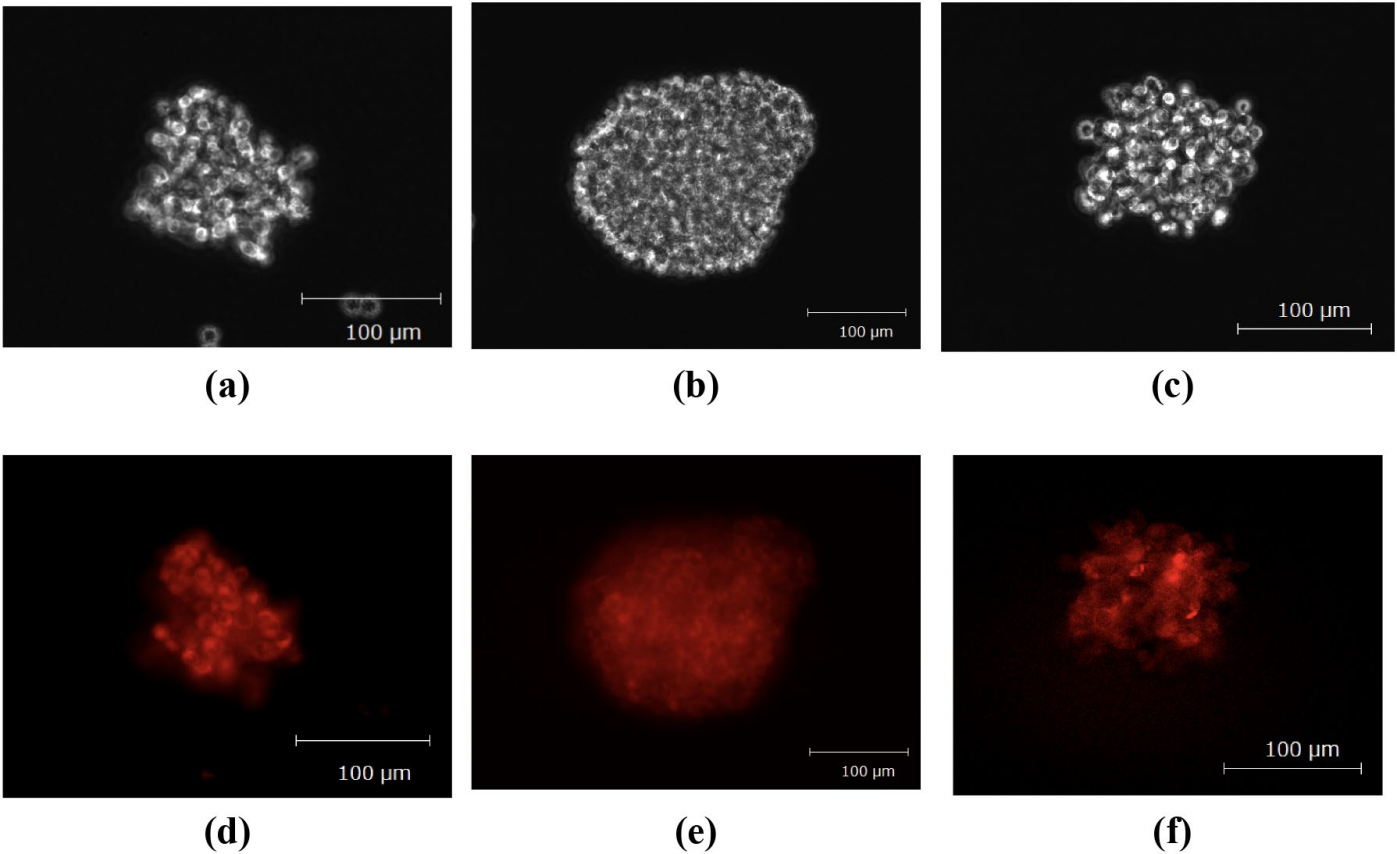
Morphology of HGG13 (**a**), HGG30 (**b**), and TS (**c**) glioma stem cells (GSCs). Upper panel shows spheroids formed by GSCs in neural stem cell culture conditions. Lower panels (**d**–**f**) show corresponding images of PpIX-fluorescence 4 h after 5-ALA (300 μΜ) exposure.

**Figure 2:**
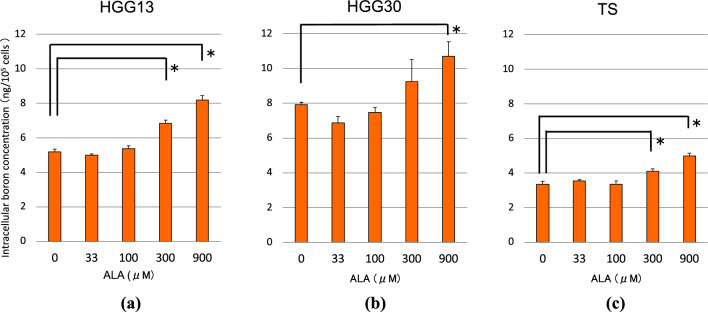
Intracellular ^10^B concentrations 6 h after BPA (1 mM) exposure. Cells were pre-incubated with ALA (0 to 900 μΜ) for 24 h. HGG13 (**a**), HGG30 (**b**), and TS (**c**) cells (*p < 0.01).

We observed cell morphology. Cell morphology observation revealed that GSCs (HGG13, HGG30, and TS) formed neurosphere-like spheroids (Fig. 1a–c). Images of PpIX-fluorescence were taken 4 h after 5-ALA (300 μΜ) exposure (Fig. 1d–f). Moreover, to examine the impact of ALA preloading on BPA uptake in GSCs, we measured the intracellular ^10^B concentration (Fig. 2a–c). ALA (300 or 900 μΜ) preloading significantly increased the BPA uptake in GSCs.

### Comparison of BPA uptake between GSCs and non-GSCs

In the absence of ALA, a significantly higher accumulation of BPA was observed in non-GSCs (HGG13/non-GSC and HGG30/non-GSC) compared to corresponding GSCs (HGG13 and HGG30); however, the difference between GSCs and non-GSCs was more apparent in HGG13 cell line (Fig. 2a–c, Supplementary Fig. S3a–c). In contrast, TS cells (mouse GSC) showed a higher accumulation of BPA than its corresponding non-GSC (TS/non-GSC) (Fig. 2a–c, Supplementary Fig. S3a–c). Based on these results, HGG13 was selected as the most appropriate cell line for in vivo experiments because it is the closest to the clinical situation.

### Expression of neutral amino acid transporters and ROS regulatory genes in human GSCs

**Figure 3:**
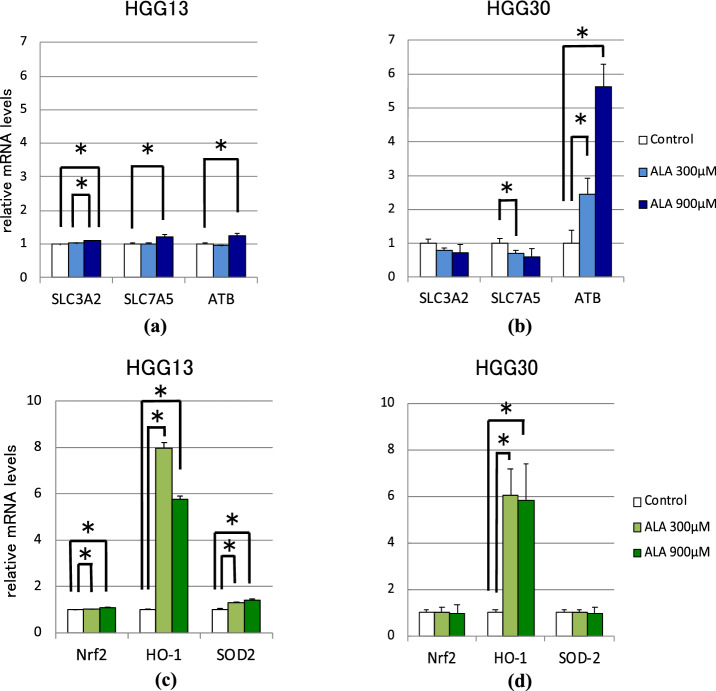
Quantitative real-time PCR analysis for evaluating the expression of neutral amino acid transporters and ROS-related genes 24 h after ALA (0–900 μΜ) exposure in human GSCs. mRNA levels of *SLC3A2*, *SLC7A5*, and *ATB*^0,+^ in HGG13 (**a**) and HGG30 (**b**). mRNA levels of *Nrf2*, *HO-1*, and *SOD2* in HGG13 (**c**) and HGG30 (d) (*p < 0.05).

We investigated the effect of ALA treatment on the expression of neutral amino acid transporters *SLC3A2*, *LAT1* (*SLC7A5*), and *ATB*^0,+^ that are reportedly involved in BPA import in human GSCs (HGG13 and HGG30) (Fig. 3a,b). Results showed that in HGG13 cells, the expression of *SLC3A2*, *SLC7A5*, and *ATB*^0,+^ was significantly increased following ALA (900 μM) pretreatment. In HGG30, *ATB*^0,+^ expression was upregulated, whereas *SLC7A5* expression was downregulated following ALA (300 μM) pretreatment.

Next, we investigated the effect of ALA treatment on the expression of *Nrf2* and Nrf2-targeted ROS scavenger genes in GSCs (Fig. 3c,d). Results showed that ALA treatment increased the levels of Nrf2, *HO-1,* and *SOD2* in HGG13. In HGG30, the expression of *HO-1* was significantly upregulated by ALA.

### Boron biodistribution in HGG13-implanted mice

Table 1 shows ^10^B concentrations in the tumor, normal brain, and blood of HGG13 glioma-bearing mice that received BPA with or without ALA pretreatment. Results showed that the mean ^10^B concentration in the tumor and T/Br ratio was higher in the ALA-pretreated group compared to the control group (23.2 vs. 20.7; *p* = 0.23 and 5.5 vs. 4.7; *p* = 0.41). However, the blood ^10^B concentration ratio was lower in the ALA-pretreated group compared to the control group (4.0 vs. 4.8; *p* = 0.23). Therefore, the T/Bl ratio was significantly higher in the ALA-pretreated group compared to the control group (5.8 vs. 4.3; *p* = 0.03).

### Evaluation of SLC3A2, LAT1 (SLC7A5), and ATB^0,+^ expression in HGG13-derived tumors

**Figure 4:**
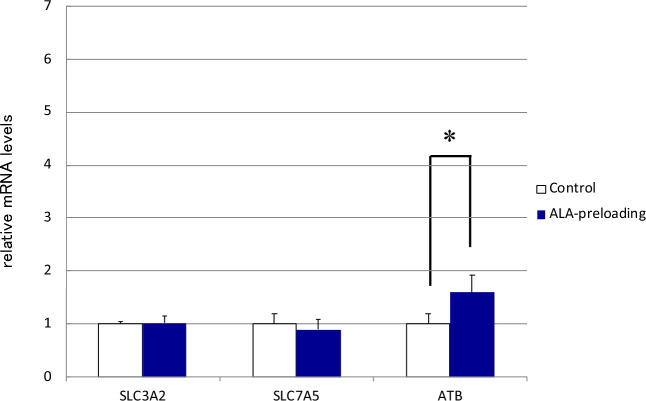
mRNA levels of neutral amino acid transporters in tumors obtained from xenograft brain tumor model mice developed using HGG13 cells. Among these transporters, only *ATB*^0,+^ expression was significantly increased in tumors isolated from the ALA-preloading group (*p < 0.01).

To validate our in vitro results, we assessed the expression of *SLC3A2*, *SLC7A5*, and *ATB*^0,+^ in tumors obtained from HGG13 glioma-implanted mice. Results showed that *ATB*^0,+^ expression was significantly increased in tumors isolated from animals that received ALA before BPA administration (Fig. 4).

### Outcomes of BNCT in HGG13-implanted mice

**Figure 5:**
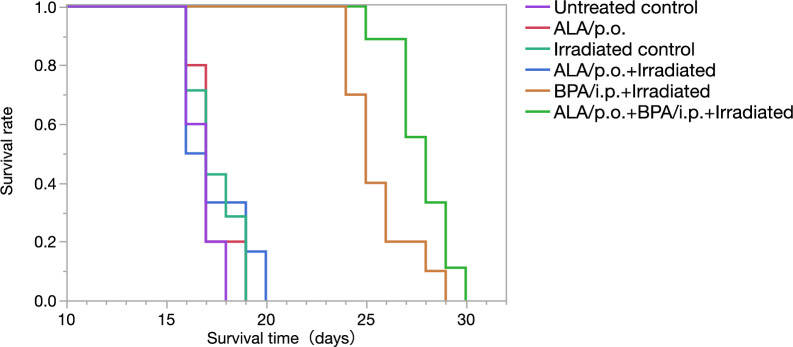
Kaplan–Meier survival curves of HGG13-bearing mice in different treatment groups. The survival time (days) after HGG13 cells implantation is shown for untreated control, ALA/p.o. only, neutron-irradiation only, Neutron irradiation following ALA/p.o., Neutron irradiation following BPA/i.p. (BNCT), Neutron irradiation following the combination of ALA/p.o. and BPA/i.p. (ALA/BNCT).

We investigated whether ALA preloading could enhance the therapeutic efficacy of BNCT using the HGG13-implanted mice model. In this experiment, one animal each in groups 4 (neutron irradiation with ALA) and 6 (BNCT with ALA), died while receiving neutron irradiation. The survival data following BNCT are summarized in Table 2. The Kaplan–Meier survival analysis is shown in Fig. 5. Results showed that the MST among the control groups (groups 1 to 4) was almost the same. However, animals in the BNCT alone group (group 5) and BNCT with ALA preloading group (group 6) showed significantly longer survival than those in the untreated control group (group 1) (*p* < 0.0001). Comparing groups 5 and 6, the survival benefit of ALA pretreatment combined with BNCT was statistically significant, but only at an approximately 8.5% increase in life expectancy (MST: 25 days vs. 28 days; *p* = 0.02).

**Table 2:** Survival time of HGG13 glioma-bearing mice following BNCT.

Group			
Untreated control	none		
ALA	ALA only		
Neutron irradiation control	Neutron irradiation only		
ALA + Neutron irradiation	Neutron irradiation following ALA		
BPA + Neutron irradiation	Neutron irradiation following BPA		
ALA + BPA + Neutron irradiation	Neutron irradiation following the combination of ALA and BPA		

Agent/route	Survival time (days)	*p*-value	
Group	Median	Vs Untreated control	Vs ALA + BPA + Neutron irradiation
Untreated control	17		< 0.0001
ALA ^a^	17	0.44	< 0.0001
Neutron irradiation control	17	0.28	< 0.0001
ALA + Neutron irradiation ^a^	16.5	0.45	< 0.0001
BPA + Neutron irradiation ^b^	25	< 0.0001	0.02
ALA + BPA + Neutron irradiation ^a, b^	28	< 0.0001	

^a^ ALA was administered orally at 80 mg/kg b.w

^b^ BPA was administered i.p. at a dose of 12 mg ^10^B/kg b.w (= 250 mg BPA/kg b.w) 24 h after ALA administration.

In vivo physical dose and equivalent dose in the tumors and the peripheral normal brain parenchyma estimated by the tissue boron concentrations are shown in Table 3.

**Table 3:** Summary of boron concentrations and physical doses and equivalent doses in GB13 glioma-bearing mice.

Group	Physical dose (Gy)^a^		Equivalent dose (Gy-Eq)^b^	
	Brain	Tumor	Brain	Tumor
Untreated control	0	0	0	0
ALA^c^	0	0	0	0
Neutron irradiation control	1.0	1.0	2.5	2.5
ALA + Neutron irradiation^c^	1.0	1.0	2.5	2.5
BPA + Neutron irradiation^d^	2.0	5.6	3.3	19.7
ALA + BPA + Neutron irradiation^c,d^	2.0	6.1	3.3	21.9

SD, Standard deviation; D_B,_ Boron dose; D_N_, neutron dose; D_H_, hydrogen dose; D_γ_, gamma-ray dose.

^a^Physical dose estimates include contributions from gamma photon, ^10^B(n,α)^7^Li, ^14^N(n,p)^14^C, and ^1^H(n,n)^1^H reactions.

^b^Equivalent dose is a value calculated using the D_B_ x CBE + D_N_ x RBE_N_ + D_H_ x RBE_H_ + D_γ_.

^c^ALA was administered orally at 80 mg/kg b.w. 24 h before BPA administration.

^d^BPA was administered i.p. at a dose of 12 mg^10^B/kg b.w (= 250 mg BPA/kg b.w).

## Discussion

GSCs are believed to be responsible for tumor relapse following standard primary therapy and poor prognosis of malignant gliomas. BNCT is a high-dose tumor-selective radiotherapy that utilizes a non-radioactive isotope of boron (^10^B) to capture thermal or epithermal neutrons. Its nuclear capture reaction yields high-LET particles^10^. Theoretically, these high-LET particles can kill both proliferating and quiescent cells, even in hypoxic microenvironments. Therefore, when an adequate amount of ^10^B is delivered to GSCs, BNCT should be able to eliminate these cells. ^18^F-BPA-PET showed that, on average, approximately 30% of BPA is taken up into the normal brain compared to the target malignant glioma, suggesting that high-dose neutron irradiation causes considerable damage to the normal brain^6^. Hence, developing novel methods to increase BPA uptake by tumor cells is necessary further to enhance the therapeutic efficacy of BNCT against malignant gliomas while maintaining its safety.

Sun et al*.* reported that culturing GSCs (SU2 cells) and non-GSCs or DGCs (SHG-44 cells) in a medium containing 5 mM BPA leads to an increase in the intracellular BPA concentration only in non-GSCs, and accordingly, GSCs showed significantly higher resistance to BNCT than non-GSCs in the clonogenic assay^11,12^. In the present work, ALA (300 or 900 μΜ) preloading significantly increased the BPA uptake in the three GSC lines under study (Fig. 2a–c).

We showed that preincubation with ALA increases the intracellular concentration of BPA in all GSC lines examined (HGG13, HGG30, and TS). In contrast, preincubation with ALA did not promote BPA accumulation in non-GSCs (Supplementary Fig. S3a–c). ALA is a hydrophilic amino acid that is hardly taken up by the normal brain tissue with an intact blood–brain barrier (BBB); however, ALA is readily taken up by malignant brain tumors with a disrupted BBB^13^. Furthermore, neoplastic cells that highly express various amino acid transporters more actively take up ALA. ALA is mainly imported into cells via peptide transporters 1 (PEPT1) and 2 (PEPT2)^14^. ALA is widely used in the fluorescence-guided diagnosis of various cancers, including malignant gliomas. The tumor-selective accumulation of ALA-derived PpIX, a fluorescent metabolite, well explains the tumor-directed nature of ALA. While PpIX fluorescence is rarely observed in tumor-adjacent normal tissues^8^.

In our previous study, we reported that the expression of PEPT1 and PEPT2 is approximately 10 times higher in GSCs compared to non-GSCs. Accordingly, GSCs accumulated significantly higher amounts of PpIX than non-GSCs upon incubation with ALA. Moreover, PpIX production in HGG13 cells is eight times higher than in the corresponding non-GSCs (HGG13/non-GSC)^15^. Based on these results, we speculate that the differences in 5-ALA uptake capacity between GSCs and non-GSCs might be a reason for the differential BPA uptake in these cells.

In all three GSC lines used in our study, the addition of ALA (900 μM) to the culture medium resulted in a significant increase in the cellular uptake of BPA (Fig. 2a–c). Previous studies suggest that the neutral amino acid transporters CD98 (SLC3A2), LAT1 (SLC7A5), LAT2 (SLC7A6), and ATB^0,+^ (SLC6A14) are involved in the transport of BPA into neoplastic cells. Wongthai et al*.* reported that among these amino acid transporters, ATB^0,+^ showed the highest rate of BPA transport in neoplastic cells^16^. Among *SLC3A2*, *SLC7A5*, and *ATB*^0,+^, the expression of *ATB*^0,+^ was significantly upregulated upon incubation with ALA in human GSCs (HGG13 and HGG30) (Fig. 3a,b). Moreover, in the HGG13-implanted glioma model, *ATB*^0,+^ was the only transporter whose expression was enhanced by ALA preloading (Fig. 4).

These results suggest that ALA-mediated increase in *ATB*^0,+^ expression might be one of the reasons for increased BPA uptake in GSCs in vitro (Fig. 2a–c). Moreover, analysis of tumors formed by the implantation of HGG13 cells into the brain revealed that intratumoral boron concentration and tumor/blood BPA ratio were higher in the ALA preloading group compared to the control group (Table 1). Compared to the in vitro study results, the ALA impact on BPA uptake was less pronounced in the in vivo experiments; in vitro studies, the cells were exposed directly to ALA. In contrast, in the in vivo experiments, ALA was administered orally, which may have had less effect on BPA uptake due to bioavailability limitations. During the post-transplant tumorigenesis process, GSCs may lose their stemness and differentiate into non-GSCs under the influence of the tissue microenvironment of the host brain. This might be one of the reasons why the results of our in vivo studies do not corroborate those of our in vitro studies. The percentage of GSCs within human glioblastomas is small, < 1%–13.6%, with the remainder being differentiated non-GSCs^17^. Our results showed that ALA significantly increases BPA uptake in GSCs but not in non-GSCs (Fig. 2a–c, Supplementary Fig. S3a–c), which explains the discrepancy between the results of our in vitro and in vivo studies.

In clinical practice, ALA has been widely used in surgery as a prodrug of the photosensitizer PpIX for fluorescence-guided resection of malignant tumors, including gliomas. Its clinical safety has already been established. Lilge et al. investigated the intracerebral distribution of ALA-induced PpIX using a rabbit brain tumor model following ALA (100 mg/kg) administration. Their results showed that the tumor PpIX concentration was approximately 10, 22, and 72 times higher than that in the peritumoral brain, normal gray matter and white matter, respectively^18^. Therefore, since ALA increases BPA uptake by tumor cells, especially GSCs, ALA might show an effect in a tumor-selective manner.

Under oxidative stress conditions, nuclear factor erythroid-derived 2 (NF-E2)-related factor (Nrf2) acts as a master switch for transcriptional upregulation of various genes coding antioxidant enzymes and transporters essential for the cellular defense against oxidative stress. These genes include *HMOX1* (*HO-1*)*, SOD2, GPX3, NQO1, SLC1A2 (GLT1), SLC1A3 (GLAST), SLC1A5 (ATB*^0^*), SLC6A5 (GLYT2),* and *SLC7A11 (XCT)*, among others^19–23^. *SLC6A14 (ATB*^0,+^*)* is also a known target gene of Nrf2^23,24^. We observed that the expression of *ATB*^0,+^ and *HO-1* was significantly higher in ALA-exposed HGG13 and HGG30 cells (Fig. 3a,c). In HGG13 cells, the expression of *Nrf2* statistically significantly increased by ALA treatment (Fig. 3c); however, in HGG30 cells, the *Nrf2* level was not affected by ALA (Fig. 3d). This suggests that the increase in *HO-1* and *ATB*^0,+^ expression in GSCs following ALA treatment is not due to increased transcript levels of *Nrf2*.

Nrf2 is a basic leucine zipper (bZIP) transcription factor that targets the antioxidant response element (ARE) with a consensus sequence of 5′-A/GTGACNNNGC^25,26^. Under normal homeostatic conditions, Nrf2 is sequestered in the cytoplasm by the E3 ubiquitin ligase Kelch ECH-associating protein 1 (Keap1) and rapidly degraded in a ubiquitin–proteasome-dependent manner^27,28^. ROS are known to oxidize two reactive cysteine residues of Keap1, inhibiting Keap1-mediated ubiquitination of Nrf2, which finally results in cytoplasmic accumulation and nuclear translocation of Nrf2^27,28^. Sugiyama et al. reported that ALA increases oxidative phosphorylation in human lung carcinoma cell line A549, increasing oxygen consumption and ROS production^29^. Furthermore, it has been reported that excess ALA can produce singlet oxygen through ALA dimerization, which leads to an increase in intracellular ROS stress^30^ and activation of Nrf2^31^. Thus, ALA inactivates Keap1 by increasing ROS levels, allowing the Nrf2 protein to accumulate in the nucleus. Within the nucleus, Bach1 is known to act as a heme-responsive transcription factor and forms a heterodimer with small Maf proteins that represses Nrf2 transcriptional activity^32,33^. It has been reported that heme can bind to Bach1, inhibiting its DNA-binding activity and promoting its nuclear export. Consequently, Nrf2 forms a heterodimer with the small Maf nuclear protein (MafK) for effective binding to the ARE consensus sequence in the promoter region of Nrf2-target genes.

During porphyrin biosynthesis, the only rate-limiting step is ALA synthesis, catalyzed by ALA synthases (ALAS1 and ALAS2). Once the intracellular heme concentration reaches optimal levels, it exerts its regulatory effect on ALAS1 and ALAS2 via negative feedback inhibition. However, exogenously imported ALA bypasses this rate-limiting step and is promptly converted to PpIX and heme without any regulation. Therefore, we speculate that excess accumulation of heme produced from exogenously supplied ALA might inactivate the Bach1 protein and promote Nrf2 binding to ARE (see Supplementary Fig. S4)^32,33^.

### Study limitations and conclusion

While culturing GSCs, we used a high concentration (20 ng/mL) of growth factors (EGF and bFGF) instead of FBS to preserve their stemness. However, these cytokines can activate porphyrin metabolism in GSCs, and consequently, GSCs might accumulate large amounts of PpIX, leading to a change in the native characteristics of these cells. Hence, there is no credible evidence that the in vitro response of GSCs to exogenous ALA observed in our study accurately represents ALA's effect on GSCs in vivo. Further studies are required to clarify whether exogenous ALA can increase the intracellular boron concentration in cancer stem cells within gliomas using in vivo glioma models.

We administered BPA in the ^10^B biodistribution study on day 14 after implantation, whereas in the BNCT experiment on day 7 after implantation. The brain tumor model used in this experiment dies from the tumor in a little over two weeks. Since removing the tumor to evaluate ^10^B biodistribution was necessary, we chose 14 days after transplantation, when the tumor should have grown to a size that could be identified by gross eye.

On the other hand, since 14 days after transplantation is the time when some animals are already on the verge of brain herniation, we decided that it would be better to conduct the BNCT experiment at 7 days after transplantation, when the tumor is smaller, in order to examine the difference in therapeutic effect among the groups. Thus, the evaluation of biodistribution after implantation and the timing of the irradiation experiment were different, so the results may not reflect the actual biodistribution at the neutron irradiation.

Finally, we performed in vivo experiments using only one GBM cell line. As GBM is highly heterogeneous, further studies using other cell lines are required to confirm whether ALA can enhance the therapeutic benefits of BNCT^34^. Further experiments will be devoted to fractionating GBM cells by trait to determine the amount of boron uptake.

In conclusion, the failure of current therapies to eliminate GSCs is considered to be one of the leading causes of the inevitable recurrence observed in patients with malignant gliomas. BNCT is a tumor-selective, high-LET radiotherapy. Therefore, it should be capable of eliminating GSCs if adequate amounts of BPA can be delivered into this low-dividing, tumor-propagating stem cells. Our data indicate that ALA could act as a drug delivery enhancer for BPA through the upregulation of the amino acid transport system in GSCs and might thus contribute to improving the therapeutic efficacy of BPA-based BNCT. Since, similarly to BPA, ALA can also selectively accumulate in neoplastic cells; it can be expected to exert a tumor-selective sensitizing effect when combined with BNCT.

## Methods

### In vitro studies

#### BPA

BPA (L-isomer) was kindly supplied by Stella Chemifa Corporation (Osaka, Japan) and was converted to fructose complex before incubation^35^. Thus, BPA used in the present study was used as a fructose complex.

#### Cell lines and cell culture

In this study, we used two human GSC lines (HGG13, HGG30) and a mouse GSC line (TS). (Fig. 1a–c). Under the institutional review board-approved protocol according to National Institute of Health (NIH) guidelines, the two human GSC lines (HGG13 and HGG30) had been established from surgical specimens of malignant gliomas after obtaining the written informed consent from the donors, as previously described^36–40^. In the present study, these human GSC lines were used after obtaining approval from the Research Ethics Committee of Osaka Medical College (Approval No.: E14-2023).

These human GSCs were classified as mesenchymal-type GSCs based on their gene expression profiles^37^. HGG13 and HGG30 cells were cultured in Dulbecco’s Modified Eagle Medium (DMEM)/F12 (Thermo Fisher Scientific, Indianapolis, IN, USA) supplemented with 20 ng/mL epidermal growth factor (EGF; PeproTech, Rocky Hill, NJ, USA), 20 ng/mL basic fibroblast growth factor (bFGF; PeproTech), B27 supplement without vitamin A (Thermo Fisher Scientific), 200 ng/mL heparan sulfate, GlutaMAX (Thermo Fisher Scientific), 100 U/mL penicillin, and 100 ng/mL streptomycin (Thermo Fisher Scientific) at 37 °C in a 5% CO_2_ atmosphere^37,41^.

TS cells are murine GSCs established by overexpressing H-RAS V12 in normal neural stem/progenitor cells isolated from the subventricular zone of adult B6 mice harboring a homozygous deletion of the INK4a/Arf locus^42^. TS cells were cultured in DMEM/F12 (Sigma, St. Louis, MO, USA) supplemented with 20 ng/mL EGF (PeproTech), 20 ng/mL bFGF (PeproTech), B27 supplement without vitamin A (Thermo Fisher Scientific), 200 ng/mL heparan sulfate, 100 U/mL penicillin, and 100 ng/mL streptomycin (Thermo Fisher Scientific) at 37 °C in a 5% CO_2_ atmosphere^42^.

#### Quantitative real-time PCR analysis for gene expression

Total RNA was extracted from cells using the RNA extraction kit (miRNeasy Mini #217,004; QIAGEN, Tokyo, Japan) and subjected to cDNA synthesis using the Transcriptor First-Strand cDNA Synthesis Kit (SuperScript VILO cDNA Synthesis Kit #11754-050; Thermo Fisher Scientific). The reaction was run in the LightCycler (Roche Diagnostics, Basel, Switzerland) according to the TaqMan assay protocol. The reaction consisted of a denaturation step (95 °C for 15 min), and the amplification and quantification process was repeated 45 times (95 °C for 15 s and 60 °C for 40 s). Data were analyzed using the LightCycler 3 software. The mRNA levels of *SLC3A2*, *LAT1* (*SLC7A5*), *ATB*^0,+^ (*SLC6A14*), *Nrf2*, *HO-1* (*HMOX1*), and *SOD2* were measured. Primer details are provided in Supplementary Table S1. Relative mRNA levels were calculated as the ratio of the target gene to GAPDH and β-actin by referring to each sample’s mRNA levels of GAPDH and β-actin.

#### In vitro uptake of BPA in GSCs

HGG13, HGG30, and TS cells were incubated with 5-aminolevulinic acid (ALA) (Cosmo Bio, Tokyo, Japan)-containing medium (0, 33, 100, 300, and 900 μΜ) for 24 h. After incubation, the cells were centrifuged and washed with phosphate-buffered saline (PBS). Cells were then exposed to 1 mM BPA-containing medium for 6 h, following which the cells were centrifuged and washed twice with PBS. The cells were then digested for 24 h with 1 N nitric acid solution (Wako Pure Chemical Industries, Osaka, Japan). The intracellular concentration of ^10^B was measured using inductively coupled plasma atomic emission spectroscopy (ICP-AES; iCAP6000 emission spectrometer, Hitachi High-Technologies, Tokyo, Japan). All the above experiments were performed under dark conditions to avoid the photodynamic effect on ALA-exposed tumor cells.

### Animal experiments

#### Animal ethics

All of the animal experiments in this study were approved by the Ethical Committee on the Care and Use of Laboratory Animals of Osaka Medical College (Approval No. 30036) and Kyoto University Research Reactor Institute (KURNS; Kumatori, Osaka, Japan) (Approval No. P10-8), respectively. Animal housing and the following animal methods and procedures were performed in accordance with the regulations and guidelines of our institutions (OMC and KURNS), both of which comply with the ARRIVE Guidelines 2.0. (PLoS Bio 8(6), e1000412,2010).

#### Mouse glioma model using a GSC line (HGG13)

Seven-week-old male BALB/c nude mice [19–23 g body weight (b.w.)] were used for the HGG13 cell transplantation (Japan SLC, Hamamatsu, Japan). All animals were raised in Specific pathogen-free (SPF)-level animal rooms and fed a standard diet. Mice were anesthetized with an intraperitoneal injection of medetomidine (0.3 mg/kg), midazolam (4 mg/kg), and butorphanol (5 mg/kg) and placed in a stereotactic frame (model 51,625; David Kopf Instruments, Tujunga, CA, USA). A midline scalp incision was made, following which a small burr hole was drilled 2 mm right lateral to the bregma using an electric drill. A 25-μL Hamilton microsyringe with a 22-gauge needle (model 1700RN, Hamilton Bonaduz, Bonaduz, Switzerland) was inserted at 4 mm depth from the skull and withdrawn from 1 mm to the target in the brain (3 mm from the skull surface). Since HGG13 cells form spheroid, the cells were dispersed by pipetting and confirmed to be single-celled using a microscope. Ten thousand HGG13 cells suspended in 2 μL of Serum-free DMEM were injected at a rate of 1 μL/min using an automatic infusion pump. The needle was placed for 2 min after infusion and withdrawn slowly. The burr hole of the skull was sealed with bone wax, and the scalp was sutured with a sterilized clip.

#### Boron biodistribution studies using the HGG13-implanted glioma model

BPA biodistribution was evaluated 14 days after HGG13 cell implantation into the mouse brain. ALA or PBS (80 mg/kg b.w.) was orally administered to mice 24 h before BPA administration. BPA (250 mg/kg b.w., equivalent to 12 mg ^10^B/kg b.w.) was administered intraperitoneally. After 2.5 h of BPA administration, the mice were euthanized, and the tumor and blood were collected to measure the tissue ^10^B concentration using ICP-AES as we described previously^43^.

#### In vivo BNCT studies using the HGG13-implanted glioma model

The in vivo BNCT study was performed using the HGG13-implanted glioma model mice at the Kyoto University Reactor (KUR) seven days after tumor cell implantation. Forty-four glioma-bearing mice were randomly divided into six groups: group 1, untreated control (n = 5); group 2, ALA-only control (n = 5); group 3, neutron irradiation-only control (n = 7); group 4, neutron irradiation following ALA (n = 6); group 5, neutron irradiation following BPA (n = 10); and group 6, neutron irradiation following the combination of ALA and BPA (n = 9).

ALA was administered orally at 80 mg/kg b.w. to animals in groups 2, 4, and 6, 24 h before BPA administration. BPA (250 mg/kg b.w., equivalent to 12 mg/kg ^10^B/kg b.w.) was administered intraperitoneally to groups 5 and 6, and PBS was administered to animals in other groups. Mice in groups 3 to 6 were irradiated at a reactor power of 1 MW at the Heavy Water Neutron Irradiation Facility (HWNIF) for 50 min, 2.5 h after BPA administration. Sham irradiation was performed on mice in groups 1 (neither BPA nor ALA) and 2 (no BPA, only ALA pre-loading). After implantation surgery, the mice’s body weight and neurological function were monitored daily. One day before death became imminent (defined by significant weight loss and a lack of activity or severe neurological deficits such as paralysis or ataxia and the onset of seizures), the mice were euthanized in accordance with the ARRIVE guidelines 2.0.

The therapeutic effects of BNCT were evaluated by measuring the survival time of glioma-bearing mice. The median survival time was calculated, and Kaplan–Meier survival curves were plotted for all groups. An overall log-rank test was performed to test the equality of the survival curves among groups.

#### Estimating of physical and equivalent doses delivered to GB13 glioma-bearing mice brain tumor model in the BNCT experiments

Estimation of the physical dose and equivalent dose delivered to the mice was done as described previously^44,45^. The BNCT dose is the sum of the physical dose attributed to the ^10^B (n, α) ^7^Li and ^14^N (n, p) ^14^C capture reactions, the ^1^H (n, n) ^1^H scattering reaction, and gamma rays, which can be described by the following equation:

1 $${\text{Physical}}\;{\text{dose}}\left( {{\text{Gy}}} \right) = {\text{DB}} + {\text{DN}} + {\text{DH}} + {\text{D}}\gamma$$

where *D*_B_ (boron dose) = 7.43 × 10^−14^ (Gy cm^2^/μg ^10^B/g) × Boron concentration (μg ^10^B/g) × thermal neutron fluence (1/cm^2^), and *D*_N_ (nitrogen dose) = 6.78 × 10^−14^ (Gy cm^2^/wt.%) × nitrogen concentration (weight%) × thermal neutron fluence (1/cm2). The above numerical values were taken from a previous study^46^. The thermal neutron fluence was measured by the radioactivity of a gold foil (0.05 mm thick, 3 mm diameter) that was set at the surface of the heads of the irradiated mice. *D*_H_ is the physical dose due to the elastic scattering between epithermal or fast neutrons and the hydrogen nucleus^47^. *D*_γ_ is the measured dose of gamma rays mixed in the neutron beam^48^. To evaluate the therapeutic effect of BNCT, each radiation dose value was multiplied by the relative biological effectiveness (RBE) for each tissue to obtain the equivalent dose, as follows:

2 $${\text{Equivalent}}\;{\text{dose}}\left( {{\text{Gy}} - {\text{Eq}}} \right) = {\text{DB}} \times {\text{CBE}} + {\text{DN}} \times {\text{RBEN}} + {\text{DH}} \times {\text{RBEH}} + {\text{D}}\gamma \left( {{\text{Gy}}} \right)$$

In BNCT, the relative biological effectiveness (RBE) is expressed as the compound biological effectiveness (CBE), which corresponds to the biological properties of the boron compounds. Its value for BPA is 3.8 for glioma and 0.9 for the brain^49^. RBE for nitrogen (RBEN) and hydrogen (RBEH) is 3.0^50^.

#### Expression of SLC3A2, LAT1 (SLC7A5), and ATB^0,+^ in HGG13-derived tumors

We examined the effect of ALA on the expression of *SLC3A2*, *SLC7A5*, and *ATB*^0,+^ in tumors derived from the HGG13 implanted glioma mice model. Fourteen days after HGG13 cell implantation, mice were administered ALA (80 mg/kg b.w.) or PBS (control). Twenty-four hours later, the mice were euthanized, and tumors were excised from the brains to extract RNA. Gene expression analysis was performed using RT-PCR.

#### Statistical analysis

In vitro data were generated from at least three independent experiments. All statistical analyses were performed using JMP Pro ver. 13 for Mac (SAS Institute Inc., Cary, NC, USA). Data are presented as mean ± SE. Comparison between two groups was performed using the Student’s t-test. A *p*-value less than 0.05 was considered statistically significant. Survival of the GSC-implanted mice was calculated for each group using the Kaplan–Meier method, and the log-rank test was used to determine the differences between survival curves. A Cox proportional hazards regression model was used to analyze the survival data, with Bonferroni’s correction for multiple comparisons.

## Supplementary Information

Supplementary Information.

## Data Availability

All data generated or analyzed during this study are included in this published article and its Supplementary Information file.

## References

[1] Stupp R (2005). Radiotherapy plus concomitant and adjuvant temozolomide for glioblastoma. N. Engl. J. Med..

[2] Bao S (2006). Glioma stem cells promote radioresistance by preferential activation of the DNA damage response. Nature.

[3] Huang Z, Cheng L, Guryanova OA, Wu Q, Bao S (2010). Cancer stem cells in glioblastoma–molecular signaling and therapeutic targeting. Protein Cell.

[4] Barth RF (2012). Current status of boron neutron capture therapy of high grade gliomas and recurrent head and neck cancer. Radiat. Oncol..

[5] Kawabata S (2011). Phase II clinical study of boron neutron capture therapy combined with X-ray radiotherapy/temozolomide in patients with newly diagnosed glioblastoma multiforme–study design and current status report. Appl. Radiat. Isot..

[6] Miyatake S (2016). Boron neutron capture therapy for malignant brain tumors. Neurol. Med. Chir. (Tokyo).

[7] Kawabata S (2021). Accelerator-based BNCT for patients with recurrent glioblastoma: A multicenter phase II study. Neurooncol. Adv..

[8] Yang X, Palasuberniam P, Kraus D, Chen B (2015). Aminolevulinic acid-based tumor detection and therapy: Molecular mechanisms and strategies for enhancement. Int. J. Mol. Sci..

[9] Hefti M (2013). Fluorescence-guided surgery for brain tumors. CNS Oncol..

[10] Barth RF, Mi P, Yang W (2018). Boron delivery agents for neutron capture therapy of cancer. Cancer Commun. (Lond)..

[11] Sun T (2012). Selective uptake of boronophenylalanine by glioma stem/progenitor cells. Appl. Radiat. Isot..

[12] Sun T (2013). Boron neutron capture therapy induces cell cycle arrest and cell apoptosis of glioma stem/progenitor cells in vitro. Radiat. Oncol..

[13] Valdés PA (2012). Gadolinium- and 5-aminolevulinic acid-induced protoporphyrin IX levels in human gliomas: An ex vivo quantitative study to correlate protoporphyrin IX levels and blood-brain barrier breakdown. J. Neuropathol. Exp. Neurol..

[14] Döring F (1998). Delta-aminolevulinic acid transport by intestinal and renal peptide transporters and its physiological and clinical implications. J. Clin. Investig..

[15] Fujishiro T (2018). 5-Aminolevulinic acid-mediated photodynamic therapy can target human glioma stem-like cells refractory to antineoplastic agents. Photodiagnosis Photodyn. Ther..

[16] Wongthai P (2015). Boronophenylalanine, a boron delivery agent for boron neutron capture therapy, is transported by ATB0,+, LAT1 and LAT2. Cancer Sci..

[17] Brescia P (2013). CD133 is essential for glioblastoma stem cell maintenance. Stem Cells.

[18] Lilge L, Wilson BC (1998). Photodynamic therapy of intracranial tissues: A preclinical comparative study of four different photosensitizers. J. Clin. Laser Med. Surg..

[19] Kobayashi A (2006). Oxidative and electrophilic stresses activate Nrf2 through inhibition of ubiquitination activity of Keap1. Mol. Cell Biol..

[20] Kobayashi M, Yamamoto M (2006). Nrf2-Keap1 regulation of cellular defense mechanisms against electrophiles and reactive oxygen species. Adv. Enzyme Regul..

[21] Moi P, Chan K, Asunis I, Cao A, Kan YW (1994). Isolation of NF-E2-related factor 2 (Nrf2), a NF-E2-like basic leucine zipper transcriptional activator that binds to the tandem NF-E2/AP1 repeat of the beta-globin locus control region. Proc. Natl. Acad. Sci. USA.

[22] Itoh K, Igarashi K, Hayashi N, Nishizawa M, Yamamoto M (1995). Cloning and characterization of a novel erythroid cell-derived CNC family transcription factor heterodimerizing with the small Maf family proteins. Mol. Cell Biol..

[23] Fledderus JO (2008). KLF2 primes the antioxidant transcription factor Nrf2 for activation in endothelial cells. Arterioscler. Thromb. Vasc. Biol..

[24] Shen G (2006). Modulation of nuclear factor E2-related factor 2-mediated gene expression in mice liver and small intestine by cancer chemopreventive agent curcumin. Mol. Cancer Ther..

[25] Motohashi H, Yamamoto M (2004). Nrf2-Keap1 defines a physiologically important stress response mechanism. Trends Mol. Med..

[26] Nguyen T, Sherratt PJ, Nioi P, Yang CS, Pickett CB (2005). Nrf2 controls constitutive and inducible expression of ARE-driven genes through a dynamic pathway involving nucleocytoplasmic shuttling by Keap1. J. Biol. Chem..

[27] Hagiya Y (2008). Nrf2-dependent induction of human ABC transporter ABCG2 and heme oxygenase-1 in HepG2 cells by photoactivation of porphyrins: Biochemical implications for cancer cell response to photodynamic therapy. J. Exp. Ther. Oncol..

[28] Ishikawa T (2013). Role of Nrf2 in cancer photodynamic therapy: Regulation of human ABC transporter ABCG2. J. Pharm. Sci..

[29] Sugiyama Y (2014). The heme precursor 5-aminolevulinic acid disrupts the Warburg effect in tumor cells and induces caspase-dependent apoptosis. Oncol. Rep..

[30] Di Mascio P (2000). DNA damage by 5-aminolevulinic and 4,5-dioxovaleric acids in the presence of ferritin. Arch. Biochem. Biophys..

[31] Loboda A, Damulewicz M, Pyza E, Jozkowicz A, Dulak J (2016). Role of Nrf2/HO-1 system in development, oxidative stress response and diseases: An evolutionarily conserved mechanism. Cell. Mol. Life Sci..

[32] Ogawa K (2001). Heme mediates derepression of Maf recognition element through direct binding to transcription repressor Bach1. EMBO J..

[33] Igarashi K, Sun J (2006). The heme-Bach1 pathway in the regulation of oxidative stress response and erythroid differentiation. Antioxid. Redox Signal..

[34] Bonavia R, Inda MM, Cavenee WK, Furnari FB (2011). Heterogeneity maintenance in glioblastoma: A social network. Cancer Res..

[35] Coderre JA (1994). Neutron capture therapy of the 9L rat gliosarcoma using the p-boronophenylalanine-fructose complex. Int. J. Radiat. Oncol. Biol. Phys..

[36] Miyazaki T (2012). Telomestatin impairs glioma stem cell survival and growth through the disruption of telomeric G-quadruplex and inhibition of the proto-oncogene, c-Myb. Clin. Cancer Res..

[37] Mao P (2013). Mesenchymal glioma stem cells are maintained by activated glycolytic metabolism involving aldehyde dehydrogenase 1A3. Proc. Natl. Acad. Sci. USA.

[38] Joshi K (2013). MELK-dependent FOXM1 phosphorylation is essential for proliferation of glioma stem cells. Stem Cells..

[39] Gu C (2013). Tumor-specific activation of the C-JUN/MELK pathway regulates glioma stem cell growth in a p53-dependent manner. Stem Cells.

[40] Hemmati HD (2013). Cancerous stem cells can arise from pediatric brain tumors. Proc. Natl. Acad. Sci. USA.

[41] Ouchi R, Okabe S, Migita T, Nakano I, Seimiya H (2016). Senescence from glioma stem cell differentiation promotes tumor growth. Biochem. Biophys. Res. Commun..

[42] Sampetrean O (2011). Invasion precedes tumor mass formation in a malignant brain tumor model of genetically modified neural stem cells. Neoplasia.

[43] Yokoyama K (2006). Pharmacokinetic study of BSH and BPA in simultaneous use for BNCT. J. Neurooncol..

[44] Futamura G (2017). Evaluation of a novel sodium borocaptate-containing unnatural amino acid as a boron delivery agent for neutron capture therapy of the F98 rat glioma. Radiat. Oncol..

[45] Kanemitsu T (2019). Folate receptor-targeted novel boron compound for boron neutron capture therapy on F98 glioma-bearing rats. Radiat. Environ. Biophys..

[46] Yamatomo N (2013). Sonoporation as an enhancing method for boron neutron capture therapy for squamous cell carcinomas. Radiat. Oncol..

[47] Hughes HG (2005). MCNP5 for proton radiography. Radiat. Prot. Dosim..

[48] Sakurai Y (2000). Characteristics of the KUR heavy water neutron irradiation facility as a neutron irradiation field with variable energy spectra. Nucl. Inst. Meth. Phys. Res. A.

[49] Ono K (2016). An analysis of the structure of the compound biological effectiveness factor. J. Radiat. Res..

[50] Suzuki M (2014). Boron neutron capture therapy outcomes for advanced or recurrent head and neck cancer. J. Radiat. Res..

