# Supplementary material for: 5-Aminolevulinic acid increases boronophenylalanine uptake into glioma stem cells and may sensitize malignant glioma to boron neutron capture therapy
Source: Sci Rep. 2023 Jun 22;13:10173. doi: 10.1038/s41598-023-37296-6 (PMC10287723; doi:10.1038/s41598-023-37296-6)
Supplement: Supplementary file 1 — Supplementary Information. [file 41598_2023_37296_MOESM1_ESM.docx]

**Supplementary information**

To examine the effect of 5-ALA on intracellular phenylalanine levels in HGG13 (human GSCs) and TS (mouse GSCs) cells, we measured phenylalanine concentration in these cells after 7 days of incubation in a medium supplemented with or without 5-ALA (300 μM) using CE-TOF/MS (capillary electrophoresis equipped with time-of-flight mass spectrometry) as described previously (Mol. Biosyst. 2011, 7, 1217–1223). Results showed that compared to untreated cells, the intracellular phenylalanine concentration was approximately 1.3 times higher in 5-ALA (300 μM) treated HGG13 and TS cells. This suggests that ALA could either increase phenylalanine uptake or decrease phenylalanine consumption in GSCs since phenylalanine is an essential amino acid that cannot be synthesized by mammalian cells (Figure S1).

| **** | **** |
| --- | --- |
| **(a)** | **(b)** |

Supplementary Fig. 1. Intracellular phenylalanine levels in human (HGG13) and mouse (TS) glioma stem cells (GSCs) treated with ALA (300 μM). Phenylalanine concentration in ALA-treated cells is shown relative to that in controls. Data are represented as mean ± SD in biologically triplicate assays.

HGG13 and HGG30 cells were implanted into the brains of BALB/c nude mice, while TS cells were implanted into the brains of C57BL/6 mice. The formed tumors were removed and dissociated to recover active tumor cells using the gentleMACS™ Dissociator (Miltenyi Biotec GmbH, Bergisch Gladbach, Germany). After 2 weeks of culture in a serum-containing medium under adherent conditions, these cells showed spindle-shaped morphology, as shown in Figure S2a-c. We termed these monolayer cells as differentiated glioma cells (DGCs, non-GSCs): HGG13/non-GSC, HGG30/non-GSC, and TS/non-GSC.

We observed cell morphology and evaluated the expression of stem cell markers in GSCs and their corresponding non-GSCs. The mRNA levels of *CD44*, Kruppel-like factor 4 (*KLF4*), *SOX2* were measured. The expression of stem cell markers *CD44*, *KLF4*, and *SOX2* was significantly higher in GSCs compared to corresponding non-GSCs (Figure S2d-f).

| 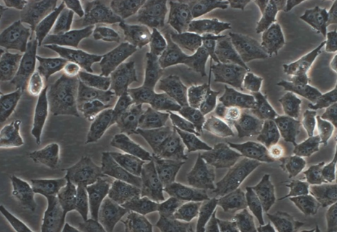 | 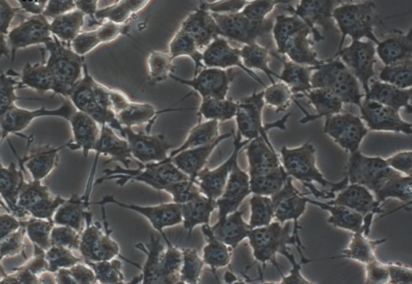 | 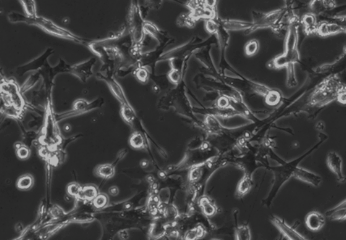 |
| --- | --- | --- |
| **(a)** | **(b)** | **(c)** |

| **** | **** |  |
| --- | --- | --- |
| **(d)** | **(e)** | **(f)** |

Supplementary Fig. 2. The upper panel shows differentiated glioma cells (DGCs) in adherent culture conditions: HGG13/non-GSC (a), HGG30/non-GSC (b), and TS/non-GSC (c). The lower panel shows the expression of stem cell markers in GSCs (red bars) and their matched non-GSCs (white bars). HGG13 (d) and HGG30 (e) cells (human GSCs), which showed mesenchymal phenotype, had significantly higher levels of *CD44* and *KLF4* than their matched non-GSCs. TS cells (f) (mouse GSC) showed high expression of *CD44* and *SOX2* compared to the corresponding non-GSC (*p < 0.01).

To examine the impact of ALA preloading on BPA uptake in GSCs and their corresponding non-GSCs, we measured the intracellular ^10^B concentration (Figure S3 a, b, and c).

| **** | **** | **** |
| --- | --- | --- |
| **(a)** | **(b)** | **(c)** |

Supplementary Fig. 3. Intracellular ^10^B concentrations 6 h after BPA (1 mM) exposure. Cells were pre-incubated with ALA (0 to 900 μΜ) for 24 h. HGG13/non-GSC (a), HGG30/non-GSC (b), and TS/non-GSC (c) cells (^#^p < 0.01)

Exogenous ALA increases intracellular reactive oxygen species (ROS) stress and is converted to heme. Under normal homeostatic conditions, Nrf2 is sequestered in the cytoplasm by Keap1 and is rapidly degraded in a ubiquitin–proteasome-dependent manner. ROS oxidizes two reactive cysteine residues on Keap1 and inhibits the ubiquitination of Nrf2, which results in both cytoplasmic accumulation and nuclear translocation of Nrf2. Under normal conditions, the transcription of ARE-downstream genes is repressed by Bach1. Heme binds to Bach1, inhibiting its DNA-binding activity and inducing its nuclear export. Thereby, Nrf2 dimerizes with the small Maf nuclear protein for effective binding to the ARE consensus sequence in the promoter region of Nrf2-target genes, including *HO-1* and *ATB^0,+^* (Figure S2).

Supplementary Fig. 4. The proposed mechanism explains the increased uptake of BPA in ALA-pretreated cells. **ABCB6**: ATP Binding Cassette Subfamily B Member 6, **ALA**: δ-aminolevulinic acid, **ARE**: Antioxidant Response Element**, ATB0+**: Solute Carrier Family 6 Member 14 (Amino Acid Transporter B0+)**, Bach1**: BTB and CNC homology 1**, BPA**: Boronophenylalanine, **HO-1**: Heme Oxygenase 1 (HMOX1)**, Keap1**: E3 ubiquitin ligase Kelch ECH-associating protein 1**, MAF**: Small musculoaponeurotic fibrosarcoma nuclear proteins**, Nrf2**: Nuclear factor erythroid-derived 2 (NF-E2)-related factor**, PEPT1**: Solute Carrier Family 15 Member 1 (Peptide Transporter 1)**, PEPT2**: Solute Carrier Family 15 Member 2 (Peptide Transporter 2)**, SOD-2**: Superoxide Dismutase 2

Supplemental Table 1. Summary of the Real-Time Polymerase Chain Reaction (PCR) Primers sequence

|  | **Gene Symbol** | **Primers** | | **Sequences** |
| --- | --- | --- | --- | --- |
| Human | GAPDH | Forward | | 5'-agccacatcgctcagacac-3' |
|  |  | Reverse | | 5'-gcccaatacgaccaaatcc-3' |
|  |  |  | |  |
|  | CD44 | Forward | | 5'-caagcaggaagaaggatggat-3' |
|  |  | Reverse | | 5'-aacctgtgtttggatttgcag-3' |
|  |  |  | |  |
|  | KLF4 | Forward | | 5'-gggagaagacactgcgtca-3' |
|  |  | Reverse | | 5'-ggaagcactgggggaagt-3' |
|  |  |  | |  |
|  | SLC3A2 | Forward | | 5'-caactaccggggtgagaact-3' |
|  |  | Reverse | | 5'-agccaaaactccagagcatc-3' |
|  |  |  | |  |
|  | SLC7A5 | Forward | | 5'-ctgctcaagccgctcttc-3' |
|  |  | Reverse | | 5'-cacgctgtagcagttcacg-3' |
|  |  |  | |  |
|  | ATB^0,+^ | Forward | | 5'-gagatttccatatctgacctacagc-3' |
|  |  | Reverse | | 5'-cagccaatgctaacataattgc-3' |
|  |  |  | |  |
|  | Nrf2 | Forward | | 5'-gagacaggtgaatttctcccaat-3' |
|  |  | Reverse | | 5'-tttgggaatgtgggcaac-3' |
|  |  |  | |  |
|  | HO-1 | Forward | | 5'-ggcagagggtgatagaagagg-3' |
|  |  | Reverse | | 5'-agctcctgcaactcctcaaa-3' |
|  |  |  | |  |
|  | SOD2 | Forward | | 5'-aagtaccaggaggcgttgg-3' |
|  |  | Reverse | | 5'-tgaacttcagtgcaggctga-3' |
| Mouse | β-actin | Forward | 5'-ctaaggccaaccgtgaaaag-3' | |
|  |  | Reverse | 5'-accagaggcatacagggaca-3' | |
|  |  |  |  | |
|  | CD44 | Forward | 5'-gcatcgcggtcaatagtagg-3' | |
|  |  | Reverse | 5'-caccgttgatcaccagctt-3' | |
|  |  |  |  | |
|  | Sox2 | Forward | 5'-ggcagagaagagagtgtttgc-3' | |
|  |  | Reverse | 5'-tcttctttctcccagcccta-3' | |
